# Implementing contingency management for stimulant use in opioid treatment programs: protocol of a type III hybrid effectiveness-stepped-wedge trial

**DOI:** 10.1186/s13012-023-01297-w

**Published:** 2023-09-13

**Authors:** Sara J. Becker, Kira DiClemente-Bosco, Kelli Scott, Tim Janssen, Sarah M. Salino, Fariha N. Hasan, Kimberly R. Yap, Bryan R. Garner

**Affiliations:** 1 Center for Dissemination and Implementation Science, Northwestern University Feinberg School of Medicine, 633 N St Clair Street, Chicago, IL 60611 USA; 2 Department of Behavioral and Social Sciences, Brown University School of Public Health, 121 S Main Street, Providence, RI 02906 USA; 3 Division of General Internal Medicine, Department of Internal Medicine, The Ohio State University College of Medicine, 2050 Kenny Road, Columbus, 43221 USA

**Keywords:** Implementation, Stepped wedge trial, Hybrid effectiveness-implementation, Substance use disorders

## Abstract

**Background:**

Contingency management (CM) is an evidence-based intervention for stimulant use and is highly effective in combination with medication for opioid use disorder. Yet, uptake of CM in opioid treatment programs that provide medication for opioid use disorder remains low. This paradox in which CM is one of the most effective interventions, yet one of the least available, represents one of the greatest research-to-practice gaps in the addiction health services field. Multi-level implementation strategies are needed to address barriers to CM implementation at both the provider- and organization-level. This type III hybrid effectiveness-implementation trial was funded by the National Institute on Drug Abuse to evaluate whether a multi-level implementation strategy, the Science of Service Laboratory (SSL), can effectively promote CM implementation in opioid treatment programs. Specific aims will test the effectiveness of the SSL on implementation outcomes (primary aim) and patient outcomes (secondary aim), as well as test putative mediators of implementation outcomes (exploratory aim).

**Methods:**

Utilizing a fully powered type III hybrid effectiveness-implementation trial with a stepped wedge design, we propose to randomize a cohort of 10 opioid treatment programs to receive the SSL across four steps. Each step, an additional 2–3 opioid treatment programs will receive the SSL implementation strategy, which has three core components: didactic training, performance feedback, and external facilitation. At six intervals, each of the 10 opioid treatment programs will provide de-identified electronic medical record data from all available patient charts on CM delivery and patient outcomes. Staff from each opioid treatment program will provide feedback on contextual determinants influencing implementation at three timepoints.

**Discussion:**

Between planning of this protocol and receipt of funding, the landscape for CM in the USA changed dramatically, with multiple Departments of Health launching state-wide CM initiatives. We therefore accelerated the protocol timeline and offered some cursory training resources to all sites as a preparation activity. We also began partnering with multiple Departments of Health to evaluate their rollout of CM using the measures outlined in this protocol.

**Trial registration:**

This study protocol is registered via ClinicalTrials.gov Identifier: NCT05702021. Date of registration: January 27, 2023.

**Supplementary Information:**

The online version contains supplementary material available at 10.1186/s13012-023-01297-w.

Contributions to the literature

- This protocol aims to implement contingency management—one of the most effective, yet least available behavioral treatments for stimulants—across 10 opioid treatment programs.
- An empirically supported, multi-level implementation strategy called the Science to Service Laboratory is used to address barriers at the provider- and organization-levels.
- This protocol uses a structured methodology for specifying, tracking, and evaluating the SSL.
- Findings have the potential to improve the quality of care in opioid treatment programs and specify the key ingredients of a multi-level implementation strategy.

## Background

Contingency management is one of the most—if not *the* most—effective interventions for stimulant use and is an evidence-based adjunct to medication for opioid use disorder (MOUD) [1, 2]. MOUD is the first-line, evidence-based treatment for opioid use disorder [3–6], but does not specifically reduce stimulant use [7], and there are not yet efficacious medications for stimulant addiction. Moreover, individuals who continue to use stimulants while receiving MOUD have worse treatment response with respect to retention and abstinence from opioids [8]. Contingency management (CM) targets stimulant use via reinforcing incentives for attaining abstinence [9]. The incredible body of evidence for CM led to the Veteran’s Administration (VA) decision to rollout CM to over 100 outpatient substance use programs serving patients with stimulant use disorder [10].

Despite its status as one of the *most* effective interventions for stimulant use, CM is one of the *least* available in community settings [11]. Surveys of front-line addiction treatment providers suggest that as few as 10% of providers use CM [12]: indeed, providers are more likely to report using confrontation than CM. This paradox—in which CM is one of the most effective, yet least implemented, interventions—reflects several distinct barriers to implementation at both provider and organizational levels.

First, OTP providers are often unfamiliar with CM. In interviews with 43 OTP providers across the state of Rhode Island, our team found that only 42% were able to define CM correctly [13]. The remaining counselors either answered “I don’t know” (42%) or provided a casual definition of CM as “incentives” (16%) without any linkage to treatment goals; such casual understanding of CM as “incentives” was consistent with findings of Rash and colleagues, which noted that what is called CM in clinical practice rarely adheres to evidence-based CM protocols [13]. Second, OTP providers often object philosophically to the idea of incentivizing patients for meeting treatment goals [14]. Our team recently surveyed 201 counselors about their attitudes towards CM prior to receipt of training and found that 16–38% disagreed with statements such as: it is appropriate for patients to earn prizes for meeting treatment goals; incentives can have a positive effective on the patient/counselor relationship; CM is an acceptable intervention, and CM is effective for patients [15]. Third, and most critically, organizational-level barriers such as funding, weak implementation climate, and limited time to learn or deliver new practices often impede successful implementation and sustainment of CM [11, 16, 17]. For example, in a study of 60 OTP providers followed for a year, our team found that those who did not implement CM cited organizational-level barriers far more often than they reported provider- or patient-level barriers [18]. Such findings are consistent with feedback from leaders within the VA initiative who attributed the success of their rollout to institutional funding and commitment to ongoing training throughout the integrated system [10]. Implementing CM in OTPs that lack the organization-level resources of the VA system, and that are characterized by high levels of turnover, pose a host of additional contextual barriers.

The Science to Service Laboratory (SSL) is a multi-level implementation strategy that has demonstrated effectiveness in OTPs [19–21]. The SSL was first developed and described by the New England Addiction Technology Transfer Center (ATTC), a regional intermediary purveyor organization that provides technical assistance to organizations across the 6-state region [19]. The SSL combines three elements supported by the extensive literature on effective implementation to address contextual determinants at multiple levels [22, 23]: didactic workshop + performance feedback + external facilitation. Didactic training and performance feedback components address provider-level determinants such as knowledge and perceptions of CM, whereas external facilitation addresses organization-level determinants such as funding, provider turnover, implementation climate, and leadership engagement.

Compelling support for the SSL was provided via a year-long quasi-experimental study in which 18 OTPs received either the SSL (7 OTPs based in New England) or didactic training alone (11 OTPs based outside of the region) to implement CM targeting attendance [20, 21]. Over 52 weeks, OTP staff receiving the SSL had higher odds of CM adoption (odds ratios = 2.4–13.5), greater speed of adoption (2–10 weeks faster adoption for any given CM target), and greater frequency of CM delivery (70% more CM sessions delivered) relative to those receiving didactic training only. Additional support for the SSL is currently being generated in a larger, more rigorous cluster randomized trial with 28 OTPs, which has provided preliminary data suggesting that OTPs receiving the SSL are adapting CM, delivering CM sessions with fidelity, and implementing CM consistently [24].

The SSL has demonstrated effectiveness in promoting the implementation of CM targeting attendance [20], but has never been evaluated for the implementation of CM targeting stimulant use. Furthermore, prior SSL studies have been subject to several limitations. First, previous investigations have confounded receipt of the SSL implementation strategy with provision of CM resources by providing OTPs a fully stocked prize cabinet at the start of implementation [20, 21, 24]. Second, SSL trials have only measured sustainment for up to 6 months [24], which limits conclusions about the ability of OTPs to continue CM implementation without active support. Finally, studies of the SSL have not rigorously tracked adherence to implementation strategy [20, 21, 24]. The current project will address these three gaps by: decoupling the SSL strategy from the provision of incentives; using a stepped wedge design that enables monitoring of sustainment over a longer time horizon; and rigorously specifying and tracking the SSL strategy.

Our protocol is guided by three specific aims:

- Specific aim 1 (primary aim): to test the effectiveness of the SSL on implementation outcomes. It is hypothesized that receipt of the SSL will be associated with significant increases in: CM Reach (percent of patients receiving CM), CM Adoption (percent of providers delivering CM), and CM Maintenance (reach and adoption after removal of active support).
- Specific aim 2 (secondary aim): to test the effectiveness of the SSL on patient outcomes. It is hypothesized that receipt of the SSL will be associated with significant increases in: Stimulant Abstinence (percent of stimulant-negative screens) and Treatment Retention (number of treatment sessions).
- Specific aim 3 (exploratory aim): to evaluate contextual determinants, including SSL adherence, as putative mediators of implementation outcomes. It is hypothesized that contextual factors at the organization-level (e.g., implementation climate, leadership) and provider-level (e.g., perceptions of CM) will partially explain the effect of the SSL on implementation (Aim 1) outcomes.

This protocol describes one of three coordinated research projects within the Center for Dissemination and Implementation at Stanford (C-DIAS), a center of excellence funded by the National Institute of Drug Abuse (P50DA054072). Each project shares a set of common implementation strategy tracking tools and implementation measures with a unified goal of promoting equitable access to evidence-based addiction treatments.

## Methods/design

### Study design

We report this protocol using the SPIRIT guidelines for reporting of intervention trials (see Additional file 1) [25]. This protocol uses a type 3 hybrid effectiveness-implementation design, which prioritizes testing the SSL implementation strategy while gathering information on the impact of CM on patient outcomes [26]. We prioritize implementation outcomes, because prior work has established robust support for CM as an effective treatment for stimulant use [27]. We use a hybrid type 3 design that includes measures of clinical effectiveness instead of an implementation trial, because successful implementation does not always result in improved patient outcomes [28, 29].

This trial uses a unidirectional crossover stepped wedge design (i.e., all sites crossover in the same direction from usual care to SSL) with 10 OTPs. A stepped wedge design is a sequential roll-out of an innovation (i.e., the SSL implementation strategy) over several discrete time points or “steps.” Hemming and Taljaard [30] recently argued that stepped wedge trials pose greater risk of misspecification than cluster randomized trials and should only be used when the stepped wedge trial meets at least one of four overlapping conditions are met: (a) provides a means to conduct a randomized evaluation that would not otherwise be feasible; (b) enhances the acceptability of a randomized evaluation to key gatekeepers; (c) is the only feasible design due to practice and logistical considerations; and (d) has increased statistical power over other study designs. This study meets all four of these conditions. We are partnering with the New England ATTC, which developed the SSL and is tasked with working with Single State Authorities to support the addiction treatment and recovery support workforce. The Single State Authorities were not amenable to randomizing OTPs to two different strategies: the Single State Authorities asked that all OTPs receive the same implementation strategy for equity reasons. Our partners at the New England ATTC also preferred to offer all agencies the same implementation strategy consistent with their usual operations.

**Fig. 1:**
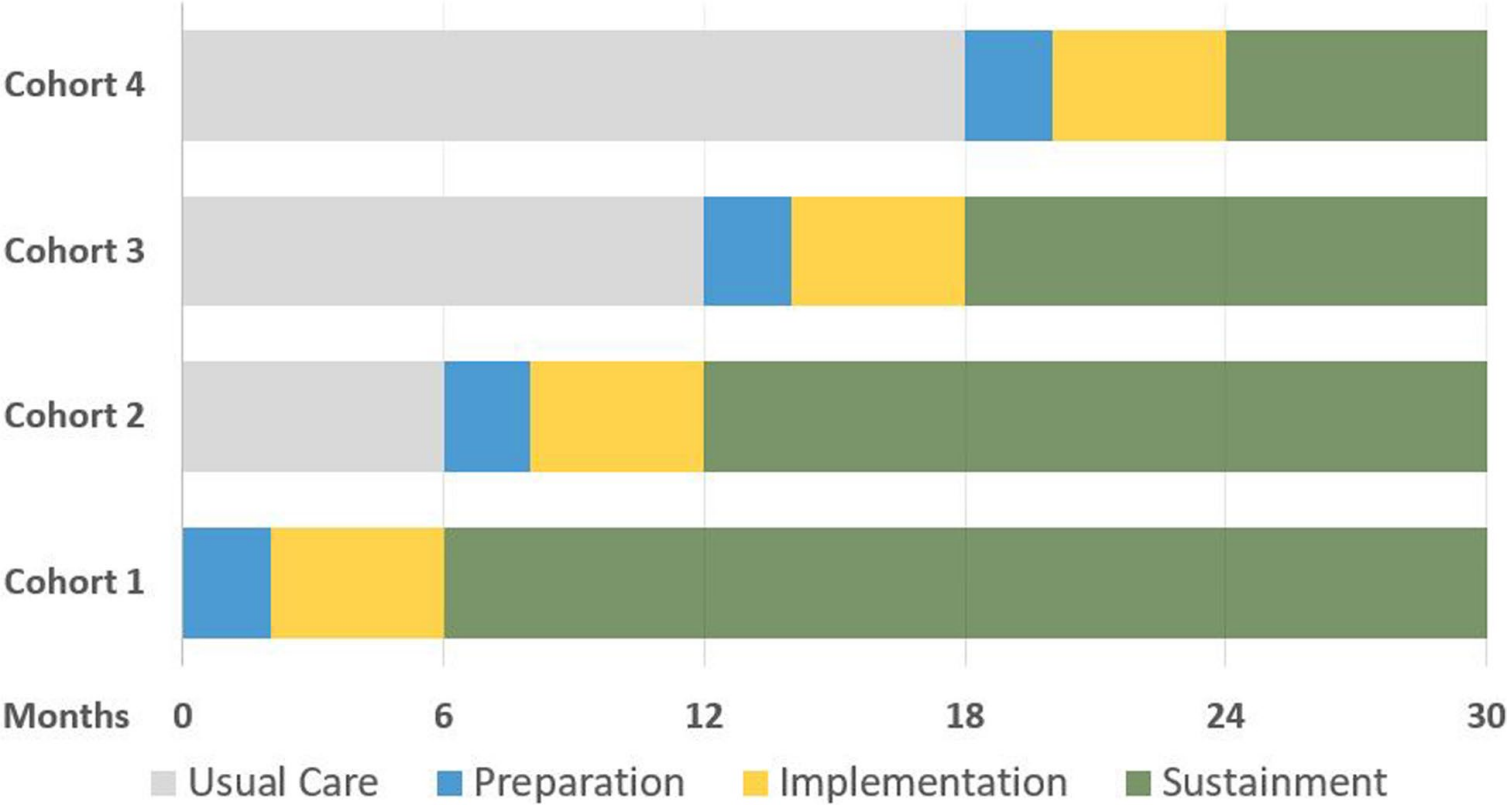
Stepped wedge design and timeline. Ten opioid treatment programs are randomized to crossover from usual care (depicted in grey) to receipt of the Science to Service Laboratory across four wedges. The Science to Service Laboratory consists of preparation activities (depicted in blue) and active implementation activities (depicted in yellow). Following 6 months of active Science to Service Laboratory support, the programs shift into a sustainment phase (depicted in green) during which they receive minimal support. Programs provide data at the 0, 6, 12-, 18-, 24-, and 30-month marks

As shown in Fig. 1, 10 OTPs crossover from implementation as usual to the SSL strategy across four discrete wedges or cohorts. At the start of each wedge, 2–3 OTPs are randomly assigned to crossover. Within each cohort, we apply the Exploration, Preparation, Implementation, Sustainment [31] process model to divide implementation activities into discrete implementation phases. Exploration activities were completed by our team in prior work designing this study [18]: the SSL strategy condition will therefore contain only Preparation, Implementation, and Sustainment activities.

During the first wedge, two OTPs will be randomly selected to crossover from usual care into the SSL implementation strategy condition. These two OTPs receive the SSL elements corresponding to the Preparation and Implementation phases of implementation. During the second wedge, the next cohort of two OTPs receives the SSL elements corresponding with the Preparation and Implementation phases and the first cohort shifts to the Sustainment phase, during which SSL activities are stopped and sustainment is tracked. The crossover pattern continues until all OTPs have received active SSL support. Randomization will be done using a random number generator prior to the start of each wedge by the study statistician. Immediately after randomization, he will inform the study team, who will commence scheduling of Preparation activities with the randomly selected sites. To ensure decoupling of financial support with the randomization to the SSL, OTPs receive a flat stipend each year that they participate in the study.

A key benefit of the stepped wedge trial is the extended timeline to measure sustainment. Over a 30-month study, the stepped wedge design allows the first wedge to provide sustainment data for up to 24 months.

### Participant recruitment

Reflecting the multi-level SSL strategy, we recruit participants at three levels.

#### Organization-level: OTPs

We will recruit 10 OTPs in New England serving a mix of urban, rural, and suburban communities, with patients from a broad array of socio-economic and racial/ethnic backgrounds. Eligible OTPs must meet the following criteria: (a) prescribe FDA-approved medication to treat patients with an opioid use disorder and concurrent stimulant use; (b) enroll at least 5 new patients per month; and (c) have at least 3 counselors who offer psychosocial support to OTP patients. We estimate that approximately 8 patients will be observed via electronic medical record (EMR) pulls per month at each OTP. Across 10 OTPs and 30 months of observation, this results in an estimated 2400 EMR pulls, across 70 counselors (assuming counselor turnover).

Research staff will recruit OTPs via established relationships with Departments of Health throughout New England. The Departments of Health host regular meetings with OTPs throughout their state and the research team will be invited to present a study overview at one of these meetings. The study overview consists of a standard set of presentation slides and a visual flyer that summarizes benefits of participation. The research team used this approach to efficiently recruit 28 OTPs in their prior cluster randomized trial.

#### Provider-level: OTP counselors and leaders

Across the 10 OTPs, all counselors and leaders (e.g., clinical supervisors, program directors) engaged in direct treatment provision or oversight will be invited to participate. Eligible counselors must: (a) provide ongoing psychosocial support to OTP patients (e.g., individual and/or group counseling sessions), and (b) have an active caseload. Eligible leaders must supervise or manage frontline CM counselors.

Once an OTP administrator indicates interest in having their organization participate in the study, the administrator will provide a list of leaders and counselors. Research staff will outreach to counselors and leaders to invite them to enroll via a combination of email, phone, or text, with follow-up visits to the OTPs as needed. Providers and leaders will be invited to receive the SSL support regardless of whether they enroll in the study: enrollment will determine whether they provide evaluation data.

#### Patient-level: OTP patients

Eligible patients will be newly initiated on MOUD (within past 30 days) at the participating OTPs and have concurrent stimulant use (indicated by self-report of stimulant use or positive toxicology screen within past 30 days). We focus on patients newly initiated on MOUD because need is highest among these patients: drop-out rates and missed doses are higher during the first 6 months in any subsequent period [5, 6, 32]. Data from these patients will be extracted from the electronic medical record.

### CM intervention

The CM protocol that will be implemented is Petry’s evidence-based low-cost CM targeting stimulant abstinence [10, 33, 34], which provides immediate reinforcement via prize draws on an escalating schedule. We chose this CM model because of literature supporting better outcomes for patients with stimulant use when: reinforcement is provided immediately [35]; abstinence is the target [36]; and incentive values escalate to promote retention and enhance the patient’s motivation over time [35, 37, 38]. We simplified Petry’s schedule based on formative research with OTP providers who requested simple, weekly draws [13] and a prize system that could be feasibly sourced (i.e., gift cards).

For this study, partner OTPs will administer one random toxicology screen to CM patients per week for 12 weeks. OTPs may choose to test other substances, but reinforcement will be contingent on a negative stimulant result. OTPs may choose which provider (e.g., nurse, counselor) will administer the screen. The CM counselor of record will review the results with the patient.

Patients will earn prize draws for each stimulant negative sample. Draws increase by one for each negative screen for 12 weeks, yielding a maximum of 78 draws for patients who submit 12 negative samples (i.e., 1 + 2 + 3 + 4 + 5 + … + 11 + 12). A refused or missed screen (i.e., unexcused absence on a testing day) resets draws for the next negative sample down to one, with draws again escalating for sustained abstinence. Patients draw prizes from fishbowls containing 500 slips of paper; 250 state “good job!” but are not associated with a prize, 209 state “small,” 40 state “large,” and one “jumbo.” Using these probabilities and magnitudes of $5, $25, and $100 for the three respective prize sizes, each draw has an average cost of $4.21. Thus, for a 12-week protocol, patients maintaining abstinence would be expected to earn an average of 78 draws × $4.21 draw = $328 in prizes. This maximum anticipated earning rate is identical to the rate used by Dr. Petry’s team in the VA rollout [10]. Of note, the average cost of CM in the VA was only $150 per patient, due to missed sessions resulting in resets back to 1 draw [10]. Ensuring OTPs can sustain this investment after removal of support is a key aspect of SSL external facilitation.

### The Science to Service Laboratory (SSL)

**Fig. 2:**
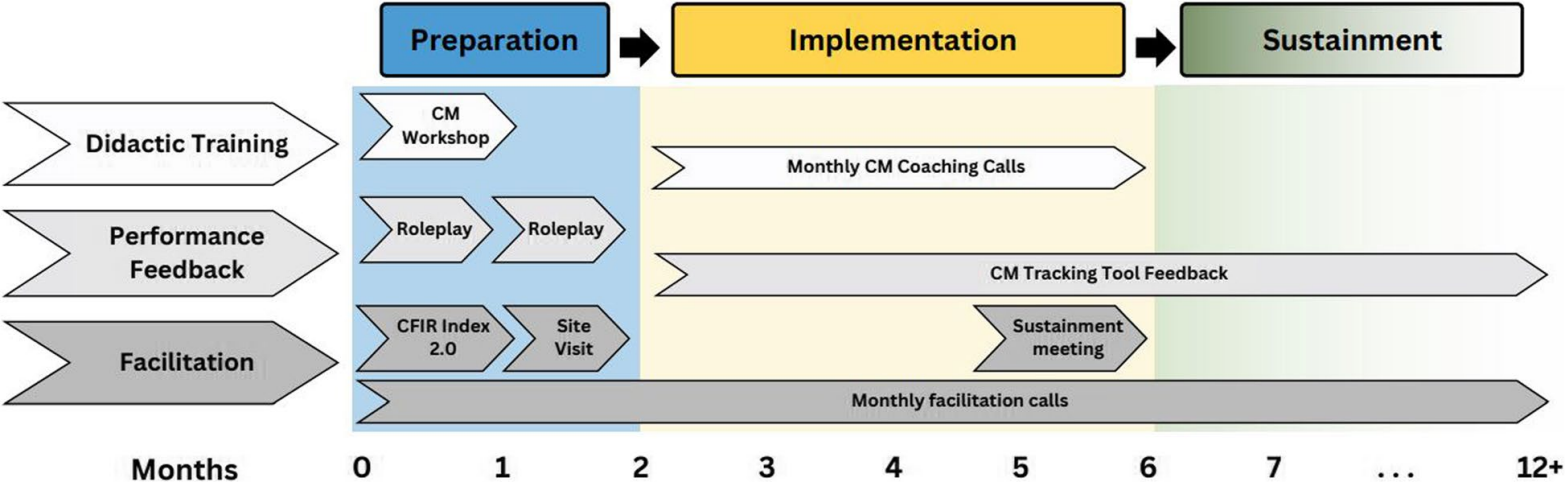
Elements of the Science to Service Laboratory

Once OTPs are randomly selected to crossover from usual care to active implementation, they will receive the SSL strategy. Detailed descriptions of each element of the SSL strategy are provided below and an overview is shown in Fig. 2.

#### Preparation phase

Each wedge begins with 2 months of Preparation activities. At the start of this phase, counselors and leaders will complete the first of three provider surveys and a team-based inventory.

##### Didactic training

A CM-delivery expert will lead a full-day didactic workshop for each OTP. The first half of the day will consist of didactic instruction in CM principles, review of videotaped exemplar CM sessions, and demonstration of CM sessions reinforcing abstinence. The second half of the day will consist of modeling CM role-plays. During the last hour of the workshop, counselors will break into pairs to complete role plays using standardized patient scenarios. Counselors will take turns playing the patient and the provider. Role plays will cover a range of possible situations in which CM counselors would have to describe CM to a new patient, provide reinforcement to a patient testing negative, and withhold reinforcement from a patient testing positive. At the end of the training, attendees will complete a 20-item CM Knowledge Scale, which requires attendees to understand and apply CM principles to case vignettes.

##### Performance feedback

The CM Competence Scale [39], a fidelity scale measuring nine key CM session elements and three general CM skills, will be provided during the didactic training along with a rating manual. This scale will be used to guide provision of performance feedback. Items are scored on 7-point Likert scales: an average score of 4.0 out of 7.0 is considered an adequate fidelity score [34].

During the preparation phase, all OTP counselors will receive performance feedback on two practice cases using the CM Competence Scale. First, the role plays at the end of the training workshop will be observed by CM experts on the research team to determine if each counselor demonstrated adequate fidelity. Live performance feedback will be provided immediately after each role play. Counselors who do not exhibit adequate fidelity during the live role-play will be given corrective feedback and the opportunity to complete another role play until each element of the CM Competence Scale is covered. Second, counselors will have 1 month following the workshop to submit an audio recording of a role-played CM session, with another CM counselor or leader at their OTP. Role plays will use a standardized patient script and be rated by blind coders. CM counselors who demonstrate satisfactory competence will be given positive performance feedback whereas those counselors who do not will be given corrective feedback. OTP leaders will be encouraged to complete fidelity ratings of two role-play sessions to gain familiarity with using the CM Competence Scale as a performance feedback tool.

Additional feedback will be provided via a 20-item CM Knowledge test. CM counselors scoring ≤ 16 (75%) on their test will receive corrective feedback. Leaders will receive a group-level summary of CM Knowledge scores by item for their OTP, to identify areas in need of further training or remediation.

##### External facilitation

External facilitation in the SSL follows the protocols outlined in the Implementation Sustainment Facilitation strategy, which has demonstrated effectiveness in a cluster randomized trial [40]. The SSL facilitator will use the publicly available facilitator workbook, meeting agendas, and worksheets for the Implementation Sustainment Facilitation strategy [41].

Throughout the Preparation phase, the SSL facilitator will hold monthly site-specific meetings to prepare for CM implementation. Topics covered in these meetings will include: discussing the goals of the implementation initiative; encouraging staff to attend training; addressing any emergent issues related to staff engagement in training; and increasing organizational preparedness for CM implementation. In addition, the facilitator will conduct an in-person site visit to engage in strategic planning for CM workflow integration. Data from the scales given at the start of the preparation phase (leadership engagement, implementation climate, CM attitudes) will be used to identify potential barriers and facilitators to CM implementation and to develop a plan to address them. The meeting will also consist of a CM process walkthrough. A major focus will be on ensuring that the necessary resources will be allocated to launch CM.

#### Implementation phase

When preparation activities are complete, OTPs shift into the active Implementation phase lasting 4 months. The goal of this phase is to commence CM implementation and integrate it into the usual workflow.

##### Didactic training

The team’s CM expert will offer monthly calls for OTPs. These calls give OTP counselors and leaders the chance to ask questions about CM techniques. Call content will focus on building CM knowledge and awareness and will not be focused on implementation.

##### Performance feedback

CM fidelity monitoring will be enhanced by the CM Tracking Tool, an Excel tracking tool developed by the study team. The tool prompts counselors to complete a brief Weekly Report for each CM patient. Each report takes 2–3 min to complete and collects date of the CM session; results of the stimulant urine screen; number of CM draws administered; and specific draws received. It also contains the first 6 items of the CM Competence Scale, which counselors complete as a self-reported fidelity check. Data input into the CM Tracking Tool yield a user-friendly dashboard for counselors that shows each patient’s progress through the CM program, summarizes fidelity of CM delivery, and automatically calculates the number of draws earned and number of draws anticipated in the next session. The CM Tracking Tool is used as the basis of a recommended EMR build for the OTPs and serves as a viable low-tech alternative.

##### External facilitation

In addition to monthly calls with a CM expert, OTP counselors and leaders will have monthly site-specific meetings with an external facilitator, following the protocols from the Implementation Sustainment Facilitation workbook [41]. Facilitation meetings are used to review each OTP’s implementation progress and discuss ways to improve performance. In the final month, the facilitator will lead a sustainment-centered planning retreat to (a) review center performance during the Implementation phase, (b) discuss the extent to which, and how, the center plans to continue CM implementation; and (c) develop a concrete sustainment action plan. Given that inadequate funding is a key barrier to CM sustainment, a primary focus of the facilitator’s work will be helping organizational leadership to evaluate a range of options to financially support CM, including soliciting prize donations, applying for grant funding, and/or applying for state funding.

#### Sustainment phase (remaining wedges)

The focus of this phase is having OTPs sustain CM without formal support. OTPs will continue to have access to the CM tracking tool and to the monthly all OTP calls with a CM expert. OTPs will also be encouraged to provide ongoing performance feedback to CM counselors as part of their ongoing operations.

#### Provider turnover plan

Throughout each stage of the SSL, we anticipate that a substantial proportion of counselors and leaders who consent will leave their OTP or the field entirely, and that new providers will come on board. Provider turnover research [42–44] estimates an annual turnover rate of approximately 30% among substance use disorder front-line counselors and 20% among OTP leadership. Our more recent experiences with OTPs recorded comparable rates: 32% of counselors turned over within our 6-month active implementation phase.

To address turnover, a key focus of the SSL strategy is on building the capacity of OTP leadership to provide ongoing support. We video- or audio-record SSL training and facilitation activities so leaders have easily transferrable, low-cost training materials, consistent with usual practices at the New England ATTC. Recommended training of replacement providers will consist of watching and/or listening to recordings of SSL activities. Most critically, CM providers will be required to conduct a role play that meets the CM Competence Scale benchmark before being approved to implement CM. Any time a role play is submitted by a replacement provider, a site leader will be encouraged to review it and submit their own CM Competence Scale rating. This will provide ongoing opportunities for leaders to gain practice rating CM sessions for fidelity and to compare ratings to those given by research staff.

### Sources of data

Data are collected via multiple methods, including EMR data extraction, provider surveys, and research team observational measures, described in detail below.

#### EMR extraction and CM tracking tool

OTPs will document CM session delivery in their usual session records within their existing EMR systems and extract a de-identified dataset including all patient encounters at six time points (start and end of each wedge). OTPs will also be given the Excel CM Tracking Tool to facilitate ongoing tracking and provision of real-time feedback, and those OTPs that opt to use the tool will be asked to send the Excel tracker at the same six time points. De-identified data from all available patient records will be identified by each site to allow the study team to calculate key study variables at each OTP including reach, effectiveness (patient abstinence and treatment retention), adoption, implementation, and maintenance.

#### Provider surveys and team-based inventory

CM providers (i.e., both counselors and leaders) will complete three online surveys to assess contextual determinants at the provider level. The provider surveys will be administered at three timepoints: at the start of each OTP’s Preparation phase, midpoint of their Implementation phase, and endpoint of their Implementation phase. In addition, CM providers at each OTP will be asked to collaboratively complete a team-based inventory of contextual determinants of implementation at the start of the Preparation phase.

#### Research team observational measures

Research staff will complete observational measures of implementation strategy progress (on an ongoing basis) and counselor fidelity to the CM model (at the end of the didactic workshop). These scales will enable us to examine the concordance between provider-self report and research staff observation.

### Study measures

To address Specific Aim 1 (implementation effectiveness), we measure the following implementation outcomes: Reach, Adoption, Implementation, Maintenance, and Equity [45]. To evaluate Specific Aim 2 (patient effectiveness), we measure two patient outcomes in line with the Effectiveness element of RE-AIM: Stimulant Abstinence and Treatment Retention. Finally, in pursuit of Specific Aim 3 (exploratory mediators), we evaluate contextual determinants as putative mediators of implementation outcomes; specifically, we assess implementation strategy progress, inner setting factors, and provider characteristics. Table 1 presents a detailed overview of study measures by Specific Aim.

**Table 1:** Description of study measures, data source, and timing of measurement

Outcome	Definition	Data source	Frequency and timing of measurement
*Aim 1*			
Reach	Calculates patients’ exposure to contingency management (CM) by dividing the number of newly admitted patients with recent stimulant use (defined as documented patient-reported stimulant use or a positive stimulant urine screen within the first 30 days of treatment) who received 1 or more CM encounters by the total number of newly admitted patients with recent stimulant use.	Electronic medical records (EMR) CM tracking tool	End of each wedge
Adoption	Calculates the proportion of providers who have adopted CM by dividing the number of counselors who delivered 1 or more CM encounters divided by the total number of counselors.	EMR CM tracking tool	End of each wedge
Implementation	Assesses delivery of CM with fidelity in two ways: 1. Proportion of counselors meeting the fidelity target on the CM Competence Scale (a mean overall score ≥ 4.0), which rates 9 CM elements on a scale from 1 (not at all present) to 7 (fully present) 2. Proportion of CM sessions covering ≥ 7 of 9 of the standard CM elements, as assessed by a self-report checklist in the CM tracking tool.	Observer rating CM tracking tool	Role play following Preparation phase End of each wedge
Maintenance	Measures Reach, Adoption, and Implementation throughout the Sustainment phase.	EMR CM tracking tool Observer rating	End of each wedge during Sustainment phase
Equity	Evaluates whether the above systematically vary based on race/ethnicity, biological sex, gender, and other sociodemographic factors of patients and providers.	EMR Provider survey	Baseline
*Aim 2*			
Stimulant abstinence	Calculates abstinence rates as the number of urine screens that are negative for stimulants divided by the total number of urine screens received by patients with recent stimulant use.	EMR	End of each wedge
Treatment retention	Measures engagement with treatment in three ways for each patient: 1. Duration of time from patient intake to discharge in day 2. Proportion of scheduled encounters that the patient attended 3. Number of CM sessions received	EMR EMR CM tracking tool	End of each wedge
*Aim 3*			
*Implementation strategy progress*			
Stages of Implementation Completion (SIC) [46]	Evaluates completion of key activities for implementing the contingency management intervention across 8 stages. Two scores are calculated for each stage: proportion calculates the percent of activities completed within a stage and duration calculates time from date of entry through completion.	Process notes External facilitator report	Ongoing throughout Preparation and Implementation phase for each OTP
Cost of Implementing New Strategies (COINS) [47]	Calculates the fees, expenses, and person hours necessary to complete the implementation strategy as mapped onto SIC. Customized via a standardized adaptation approach.	Process notes External facilitator report	Ongoing throughout Preparation and Implementation phase for each OTP
*Inner Setting Factors*			
Implementation Climate Scale (ICS) [48]	Measures provider perceptions of the extent to which organizations expect, reward, and support implementation of CM. Scale includes 18 items scored on 5-point scales from 0 (not at all) to 4 (very great extent) divided into 6 subscales.	Provider surveys	Three timepoints for each OTP crossing into active support: Start of Preparation, Midpoint of Implementation, End of Implementation
Implementation Leadership Scale (ILS) [49]	Measures providers’ report of the extent to which leaders at their organization are aligned and visibly in support of CM. Scale includes 12 items, rated from 0 (not at all) to 4 (very great extent) divided into four subscales.	Provider surveys	Three timepoints for each OTP crossing into active support: Start of Preparation, Midpoint of Implementation, End of Implementation
Inventory of Factors Affecting Successful Implementation and Sustainment (IFASIS)	Assesses barriers and facilitators to implementation via a team-based inventory collaboratively completed by each OTP at a leadership team. Consists of 27 items each scored in two ways: rating, scored on a 5-point scale ranging from 1 (least positive) to 5 (most positive), and importance, scored on a 3-point scale ranging from 1 (not important) to 3 (very important).	Recorded by leadership team	Three timepoints for each OTP crossing into active support: Start of Preparation, Midpoint of Implementation, End of Implementation
*Provider characteristics*			
AIM, IAM, FIM [50]	Measures the extent to which providers view CM as acceptable (Acceptability of Intervention Measure [AIM]), appropriate (Intervention Appropriateness Measure [IAM]), and feasible (Feasibility of Intervention Measure [FIM]). Each measure contains 4 items scored on a 5-point scale ranging from 1 (completely disagree) to 5 (completely agree).	Provider surveys	Three timepoints for each OTP crossing into active support: Start of Preparation, Midpoint of Implementation, End of Implementation
CM Attitudes Scale	Measures provider attitudes and objections towards CM via a 5-item survey; three items range from 1 (strongly disagree) to 7 (strongly agree), one item ranges from 1 (complete unacceptable) to 7 (very acceptable), and the final item ranges from 1 (not at all effective) to 7 (very effective).	Provider surveys	Three timepoints for each OTP crossing into active support: Start of Preparation, Midpoint of Implementation, End of Implementation
Provider demographics	Assesses a range of sociodemographic variables, including sex/gender, race/ethnicity, age, tenure at the OTP, licensure status, and years in the addiction health services field.	Provider survey	Baseline

### Data analysis

Across 2400 EMR pulls, 720, 480, and 1200 are expected to occur during Usual Care, Preparation/Implementation, and Sustainment phases, respectively. Preliminary analysis will include bivariate and multivariate analysis using correlations, cross-tabulations and regression models to ascertain data structure, statistical distribution, and study artefacts. Analyses for all Aims will be performed using multilevel structural equation models (MSEM) in Mplus 8.10 [51]. We conducted Monte Carlo simulation power analyses modeling treatment effects and outcome variance nested in OTPs (anticipated ICC: 15%) and/or counselors (anticipated ICC: 10%; see Table 2). There is no expected missing data from EMR pulls, although at OTP and counselor levels, analyses will follow an intent-to-treat approach to dealing with missing data. Across all analyses, we will employ a moderator-first approach to the equity of treatment effects to ensure that group-level differences can be validly interpreted and that we are sensitive to differential treatment effects among patient subgroups. Similarly, we will examine whether significant variance in treatment effects occurs at counselor- or OTP-levels (through exploring the size of random effects variance parameters) before concluding whether treatment effects can be validly interpreted as fixed at the patient-level.

**Table 2:** Results of Monte Carlo simulation analyses examining power to detect aim 1 and aim 2 outcome effects

Outcome	Comparison (*n* per condition)	Predicted rates/effects	Power at α = .05
*Aim 1*			
Reach^a^	Usual care (720)–preparation/implementation (480)	2–22%	98%
Adoption^b^	Usual care (70)–preparation/implementation (70)	10–50%	95%
Implementation	No comparison		
Maintenance	Preparation/implementation (480)–sustainment (1200)		
*Aim 2*			
Stimulant abstinence	Usual care (720)–preparation/implementation (480)	25–40%	90%
Treatment retention	Usual care (720)–preparation/implementation (480)	9 weeks–12 weeks	94%

Monte Carlo simulation multilevel power analyses featuring clustering in opioid treatment programs (anticipated ICC: 15%) and/or counselors (anticipated ICC: 10%)

^a^Assessed among patients nested in counselors and opioid treatment programs

^b^Asssessed among counselors nested in opioid treatment programs

Aim 1 hypotheses (effect of SSL on CM Reach and CM Adoption) will be tested by examining the effect of treatment condition at a given center at a given time point, for patients nested in counselors in centers (3-level model). Generalized linear mixed models will be used to evaluate treatment effects on rates of CM Reach, Adoption, and Maintenance.

For Aim 2 analyses, generalized MSEM models will be used to model the effect of SSL implementation. Using data from prior CM trials, we assume a rate increase for Stimulant Abstinence from 25 to 40% following SSL receipt, and a treatment retention increase of 30% from 9 to 12 weeks following SSL.

For Exploratory Aim 3 analyses, MSEM models will be used to assess whether putative provider-level mediators that will be assessed via the provider surveys (e.g., implementation climate, implementation leadership, CM perceptions) are associated with change in implementation outcomes. Exploratory outcome analyses will measure putative mediators as latent factors combining measures of SSL fidelity, inner setting factors, and provider characteristics, to assess the extent to which latent factors serve as mechanisms of action for the main effect of the SSL on implementation outcomes using bootstrapped confidence intervals.

### Trial status

No organizations or providers have been officially enrolled yet; recruitment is slated to begin in January 2024.

## Discussion

### Changes to the national landscape in between peer review of this proposal and funding

The national landscape surrounding CM has shifted dramatically from the time the project was originally proposed to when the project was funded. In the last 2 years, CM began making national headlines, including a New York Times article about the CM research-to-practice gap in Fall 2021 titled, “This addiction treatment works. Why is it so underutilized?” [52] and an Associated Press feature titled, “Candy, cash, gifts: How rewards help recovery from addiction” [53]. Moreover, after decades of advocacy, the state of California has decided to pilot CM as a Medicaid reimbursable service, an initiative that is being watched closely by other Departments of Health across the country. At the same time, states across the country have started to receive significant funding to address the overdose crisis via both the Substance Abuse and Mental Health Services Administration (SAMHSA) and the federal opioid settlement; reflecting the national enthusiasm for CM, some SAMHSA grant mechanisms have explicitly referenced a need for all grantees to receive CM training in the first year of funding. The more favorable national landscape for CM has affected the approach of many Departments of Health, engendering a strong desire to provide CM training to OTPs across the state as quickly as possible.

For this reason, when we connected with several Departments of Health to launch recruitment for this study, we were met with resistance to OTPs being randomized to a delayed training date. Specifically, Departments of Health noted that they were required to offer CM training to OTPs in their states and that they were not comfortable withholding training for an extended period. At that point, our team was faced with a critical conundrum that frequently plagues implementation scientists: how to balance rigorous research design with real-world priorities.

We ultimately made two changes to ensure the stepped-wedge trial would be acceptable to our partners. First, we substantially condensed the study timeline so that the longest an OTP would need to wait for intensive support would be 2 years. We had initially proposed a stepped wedge trial spanning 60 months (a timeline considered acceptable by our partners when we wrote the protocol, but far too slow once the protocol was funded), and we refined our timeline to 30 months. Second, we agreed to provide all OTPs participating in the study a 1-h self-paced webinar on CM immediately after the study start-up phase, to satisfy SAMHSA grant requirements and address the needs of our Department of Health partners. When we wrote the initial proposal, we presumed that OTPs would not have received any CM training; we now more realistically expect OTPs to have received basic CM education, as expected by SAMHSA and other state funders. This design will enable us to evaluate whether the full SSL strategy is superior to real-world implementation as usual, since CM webinars and brief training encounters are becoming more commonplace. In our experience working with the New England ATTC, organizations are highly unlikely to successfully implement a behavioral intervention like CM after a brief training encounter (please see our recent commentary, in which we noted the insufficiency of “one shot training” for greater discussion of this issue [15]); therefore, we do not expect the provision of this webinar to have any notable effect on our power assumptions or sample size calculations.

### Potential impact of protocol

Results of the current protocol have the potential to improve the quality of care in OTPs. Lessons learned may inform future implementation of evidence-based behavioral treatments in OTPs, a treatment setting that serves high-need, under-served patients at high risk of lethal overdose. Information gained may also inform efforts to improve CM implementation in other critical public health settings where CM has demonstrated effectiveness (e.g., HIV/AIDS care, prenatal care) [54, 55]. Given the dearth of knowledge on why and how implementation strategies work, our focus on specification and evaluation of contextual determinants will advance the scientific study of methods to integrate research findings and evidence-based interventions into healthcare practice and policy.

The impact of this study will be heightened by it being situated within a center of excellence in which all three major research components use common implementation strategy tracking tools and implementation outcomes. In addition, two Departments of Health have asked our team to partner with them to evaluate their state-wide efforts to fund CM implementation; we will strategically use the SSL strategy, the same implementation adherence tools, and the same set of implementation and patient outcomes to enable comparisons across initiatives. By carefully specifying and tracking the SSL strategy across these companion initiatives, our methods will help to unpack the “black box” of implementation strategy selection and deployment that so often plagues implementation science research.

### Supplementary Information

**Additional file 1.**

## Data Availability

Data collected as part of this study protocol, as well as the Data Safety Monitoring Plan, can be requested from the contact Principal Investigator, Sara Becker.

## Abbreviations

CM: Contingency management
OTP: Opioid treatment program
SSL: Science to Service Laboratory
VA: Veteran’s Administration

## References

[1] Bolivar HA, Klemperer EM, Coleman SRM, DeSarno M, Skelly JM, Higgins ST (2021). Contingency management for patients receiving medication for opioid use disorder: a systematic review and meta-analysis. JAMA Psychiat.

[2] Griffith JD, Rowan-Szal GA, Roark RR, Simpson DD (2000). Contingency management in outpatient methadone treatment: a meta-analysis. Drug Alcohol Depend.

[3] Resnick RB, Galanter M, Pycha C, Cohen A, Grandison P, Flood N (1992). Buprenorphine: an alternative to methadone for heroin dependence treatment. Psychopharmacol Bull.

[4] Mattick RP, Breen C, Kimber J, Davoli M. Methadone maintenance therapy versus no opioid replacement therapy for opioid dependence. Cochrane Database Syst Rev. 2009(3):CD002209. 10.1002/14651858.CD002209.pub2. Accessed 4 Sept 2023. 10.1002/14651858.CD002209.pub2 PMC7097731 19588333

[5] Krupitsky E, Nunes EV, Ling W, Illeperuma A, Gastfriend DR, Silverman BL (2011). Injectable extended-release naltrexone for opioid dependence: a double-blind, placebo-controlled, multicentre randomised trial. Lancet.

[6] Ling W, Casadonte P, Bigelow G, Kampman KM, Patkar A, Bailey GL (2010). Buprenorphine implants for treatment of opioid dependence: a randomized controlled trial. JAMA.

[7] Schottenfeld RS, Chawarski MC, Pakes JR, Pantalon MV, Carroll KM, Kosten TR (2005). Methadone versus buprenorphine with contingency management or performance feedback for cocaine and opioid dependence. Am J Psychiatry.

[8] Tsui JI, Mayfield J, Speaker EC, Yakup S, Ries R, Funai H (2020). Association between methamphetamine use and retention among patients with opioid use disorders treated with buprenorphine. J Subst Abuse Treat.

[9] Petry NM (2006). Contingency management treatments. Br J Psychiatry.

[10] Petry NM, DePhilippis D, Rash CJ, Drapkin M, McKay JR (2014). Nationwide dissemination of contingency management: the Veterans Administration initiative. Am J Addict.

[11] Hartzler B, Donovan DM, Tillotson CJ, Mongoue-Tchokote S, Doyle SR, McCarty D (2012). A multilevel approach to predicting community addiction treatment attitudes about contingency management. J Subst Abuse Treat.

[12] McGovern MP, Fox TS, Xie H, Drake RE (2004). A survey of clinical practices and readiness to adopt evidence-based practices: dissemination research in an addiction treatment system. J Subst Abuse Treat.

[13] Becker SJ, Scott K, Murphy CM, Pielech M, Moul SA, Yap KR (2019). User-centered design of contingency management for implementation in opioid treatment programs: a qualitative study. BMC Health Serv Res.

[14] Kirby KC, Benishek LA, Dugosh KL, Kerwin ME (2006). Substance abuse treatment providers’ beliefs and objections regarding contingency management: Implications for dissemination. Drug Alcohol Depend.

[15] Becker SJ, DiClemente-Bosco K, Rash CJ, Garner BR. Effective, but underused: lessons learned implementing contingency management in real-world practice settings in the United States. Prev Med. 2023:107594. Online ahead of print. 10.1016/j.ypmed.2023.107594 PMC10753028 37385413

[16] Walker R, Rosvall T, Field CA, Allen S, McDonald D, Salim Z (2010). Disseminating contingency management to increase attendance in two community substance abuse treatment centers: lessons learned. J Subst Abuse Treat.

[17] Ducharme LJ, Knudsen HK, Roman PM, Johnson JA (2007). Innovation adoption in substance abuse treatment: exposure, trialability, and the clinical trials network. J Subst Abuse Treat.

[18] Becker SJ, Kelly LM, Kang AW, Escobar KI, Squires DD (2019). Factors associated with contingency management adoption among opioid treatment providers receiving a comprehensive implementation strategy. Subst Abus.

[19] Squires DD, Gumbley SJ, Storti SA (2008). Training substance abuse treatment organizations to adopt evidence-based practices: the addiction technology transfer center of New England science to service laboratory. J Subst Abuse Treat.

[20] Becker SJ, Squires DD, Strong DR, Barnett NP, Monti PM, Petry NM (2016). Training opioid addiction treatment providers to adopt contingency management: a prospective pilot trial of a comprehensive implementation science approach. Subst Abus.

[21] Helseth SA, Janssen T, Scott K, Squires DD, Becker SJ (2018). Training community-based treatment providers to implement contingency management for opioid addiction: time to and frequency of adoption. J Subst Abuse Treat.

[22] Miller WR, Yahne CE, Moyers TB, Martinez J, Pirritano M (2004). A randomized trial of methods to help clinicians learn motivational interviewing. J Consult Clin Psychol.

[23] Sholomskas DE, Syracuse-Siewert G, Rounsaville BJ, Ball SA, Nuro KF, Carroll KM (2005). We don’t train in vain: a dissemination trial of three strategies of training clinicians in cognitive-behavioral therapy. J Consult Clin Psychol.

[24] Becker SJ, Murphy CM, Hartzler B, Rash CJ, Janssen T, Roosa M (2021). Project MIMIC (Maximizing Implementation of Motivational Incentives in Clinics): a cluster-randomized type 3 hybrid effectiveness-implementation trial. Addict Sci Clin Pract.

[25] Chan AW, Tetzlaff JM, Gotzsche PC, Altman DG, Mann H, Berlin JA (2013). SPIRIT 2013 explanation and elaboration: guidance for protocols of clinical trials. BMJ.

[26] Curran GM, Bauer M, Mittman B, Pyne JM, Stetler C (2012). Effectiveness-implementation hybrid designs: combining elements of clinical effectiveness and implementation research to enhance public health impact. Med Care.

[27] Stitzer ML, Petry NM, Peirce J (2010). Motivational incentives research in the national drug abuse treatment clinical trials network. J Subst Abuse Treat.

[28] Garner BR (2009). Research on the diffusion of evidence-based treatments within substance abuse treatment: a systematic review. J Subst Abuse Treat.

[29] Garner BR (2017). The relationship between several mechanism of change measures and an independently rated measure of implementation integrity.

[30] Hemming K, Taljaard M (2020). Reflection on modern methods: when is a stepped-wedge cluster randomized trial a good study design choice?. Int J Epidemiol.

[31] Moullin JC, Dickson KS, Stadnick NA, Rabin B, Aarons GA (2019). Systematic review of the Exploration, Preparation, Implementation, Sustainment (EPIS) framework. Implement Sci.

[32] Comer SD, Sullivan MA, Yu E, Rothenberg JL, Kleber HD, Kampman K (2006). Injectable, sustained-release naltrexone for the treatment of opioid dependence: a randomized, placebo-controlled trial. Arch Gen Psychiatry.

[33] Petry NM, Weinstock J, Alessi SM (2011). A randomized trial of contingency management delivered in the context of group counseling. J Consult Clin Psychol.

[34] Petry NM, Alessi SM, Ledgerwood DM (2012). Contingency management delivered by community therapists in outpatient settings. Drug Alcohol Depend.

[35] Petry N. Contingency management for substance abuse treatment: a guide to implementing this evidenced-based practice. New York: Routledge; 2012.

[36] Petry NM, Alessi SM, Carroll KM, Hanson T, MacKinnon S, Rounsaville B (2006). Contingency management treatments: reinforcing abstinence versus adherence with goal-related activities. J Consult Clin Psychol.

[37] Petry NM, Alessi SM, Marx J, Austin M, Tardif M (2005). Vouchers versus prizes: contingency management treatment of substance abusers in community settings. J Consult Clin Psychol.

[38] Lussier JP, Heil SH, Mongeon JA, Badger GJ, Higgins ST (2006). A meta-analysis of voucher-based reinforcement therapy for substance use disorders. Addiction.

[39] Petry NM, Alessi SM, Ledgerwood DM, Sierra S (2010). Psychometric properties of the contingency management competence scale. Drug Alcohol Depend.

[40] Garner BR, Gotham HJ, Chaple M, Martino S, Ford Ii JH, Roosa MR (2020). The implementation and sustainment facilitation strategy improved implementation effectiveness and intervention effectiveness: results from a cluster-randomized, type 2 hybrid trial. Implement Res Pract.

[41] Garner BR. ISF strategy: implementation and sustainment facilitation; Available from: https://www.isfstrategy.org/.

[42] Garner BR, Hunter BD, Modisette KC, Ihnes PC, Godley SH (2012). Treatment staff turnover in organizations implementing evidence-based practices: turnover rates and their association with client outcomes. J Subst Abuse Treat.

[43] Garner BR, Hunter BD (2013). Examining the temporal relationship between psychological climate, work attitude, and staff turnover. J Subst Abuse Treat.

[44] Garner BR, Hunter BD, Godley SH, Godley MD (2012). Training and retaining staff to competently deliver an evidence-based practice: the role of staff attributes and perceptions of organizational functioning. J Subst Abuse Treat.

[45] Glasgow RE, Harden SM, Gaglio B, Rabin B, Smith ML, Porter GC (2019). RE-AIM planning and evaluation framework: adapting to new science and practice with a 20-year review. Front Public Health.

[46] Chamberlain P, Brown CH, Saldana L (2011). Observational measure of implementation progress in community based settings: the Stages of Implementation Completion (SIC). Implement Sci.

[47] Saldana L, Chamberlain P, Bradford WD, Campbell M, Landsverk J (2014). The Cost of Implementing New Strategies (COINS): a method for mapping implementation resources using the stages of implementation completion. Child Youth Serv Rev.

[48] Ehrhart MG, Aarons GA, Farahnak LR (2014). Assessing the organizational context for EBP implementation: the development and validity testing of the Implementation Climate Scale (ICS). Implement Sci.

[49] Lyon AR, Cook CR, Brown EC, Locke J, Davis C, Ehrhart M (2018). Assessing organizational implementation context in the education sector: confirmatory factor analysis of measures of implementation leadership, climate, and citizenship. Implement Sci.

[50] Weiner BJ, Lewis CC, Stanick C, Powell BJ, Dorsey CN, Clary AS (2017). Psychometric assessment of three newly developed implementation outcome measures. Implement Sci.

[51] Muthén LK, Muthén B. Mplus Version 8 User's Guide. Muthén & Muthén, editors. 8th ed. Los Angeles: Muthén & Muthén; 2017.

[52] Goodnough A. This addiction treatment works. Why is it so underused?: New York Times; 2020. Available from: https://www.nytimes.com/2020/10/27/health/meth-addiction-treatment.html.

[53] Johnson C. Candy, cash, gifts: How rewards help recovery from addiction: Associated Press; 2022. Available from: https://apnews.com/article/how-rewards-helps-recovery-from-addiction-6d11673c55fae3a413dcc5f57ca5e104.

[54] Ondersma SJ, Svikis DS, Lam PK, Connors-Burge VS, Ledgerwood DM, Hopper JA (2012). A randomized trial of computer-delivered brief intervention and low-intensity contingency management for smoking during pregnancy. Nicotine Tob Res.

[55] Moore BA, Rosen MI, Wang Y, Shen J, Ablondi K, Sullivan A (2015). A remotely-delivered CBT and contingency management therapy for substance using people with HIV. AIDS Behav.

